# Pipeline Embolization Device for intracranial aneurysms presenting with mass effect: a large Chinese cohort

**DOI:** 10.1136/svn-2022-002213

**Published:** 2023-06-09

**Authors:** Yang Zhao, Junlin Lu, Hongqi Zhang, Tianxiao LI, Donglei Song, Sheng Guan, Aisha Maimaitili, Yunyan Wang, Wenfeng Feng, Yang Wang, Jieqing Wan, Guohua Mao, Huaizhang Shi, Xinjian Yang, Jianmin Liu, Yuanli Zhao

**Affiliations:** 1 Department of Neurosurgery, Beijing Tiantan Hospital, Capital Medical University, Beijing, China; 2 Department of Neurosurgery, Peking University International Hospital, Beijing, China; 3 Department of Neurosurgery, West China Hospital, Sichuan University, Chengdu, Sichuan, China; 4 Department of Neurosurgery, Xuanwu Hospital, Capital Medical University, Beijing, China; 5 Department of Interventional Neuroradiology, Zhengzhou University People's Hospital, Zhengzhou, Henan, China; 6 Department of Neurosurgery, Shanghai Donglei Brain Hospital, Shanghai, China; 7 Department of Interventional Neuroradiology, The First Affiliated Hospital of Zhengzhou University, Zhengzhou, Henan, China; 8 Department of Neurosurgery, The First Affiliated Hospital of Xinjiang Medical University, Urumqi, Xinjiang, China; 9 Department of Neurosurgery, Qilu Hospital of Shandong University Qingdao, Jinan, Shandong, China; 10 Department of Neurosurgery, Nanfang Hospital, Southern Medical University, Guangzhou, Guangdong, China; 11 Department of Neurosurgery, The First Affiliated Hospital of Nanchang University, Nanchang, Jiangxi, China; 12 Department of Neurosurgery, Renji Hospital, School of Medicine, Shanghai Jiao Tong University, Shanghai, China; 13 Department of Neurosurgery, The Second Affiliated Hospital of Nanchang University, Nanchang, Jiangxi, China; 14 Department of Neurosurgery, The First Affiliated Hospital of Harbin Medical University, Harbin, Heilongjiang, China; 15 Department of Neurosurgery, Changhai Hospital, Naval Medical University, Shanghai, China

**Keywords:** aneurysm, flow diverter, risk factors, stents

## Abstract

**Background:**

Unruptured intracranial aneurysm treatment aims to reduce the risk of aneurysm rupture and bleeding, relieves symptoms and improve the quality of life for patients. This study aimed to assess the safety and efficacy of Pipeline Embolization Device (PED, Covidien/Medtronic, Irvine, CA) treatment for intracranial aneurysms presenting with mass effect in real-world settings.

**Methods:**

We selected patients from the PED in China Post-Market Multi-Center Registry Study with mass effect presentation. The study endpoints included postoperative mass effect deterioration and mass effect relief at follow-up (3–36 months). We conducted multivariate analysis to identify factors associated with mass effect relief. Subgroup analyses by aneurysm location, size and form were also performed.

**Results:**

This study included 218 patients with a mean age of 54.3±11.8 years and a female predominance of 74.0% (162/218). The postoperative mass effect deterioration rate was 9.6% (21/218). During a median follow-up period of 8.4 months, the mass effect relief rate was 71.6% (156/218). Notably, immediate aneurysm occlusion following treatment was significantly associated with mass effect relief (OR 0.392, 95% CI, 0.170 to 0.907, p=0.029). Subgroup analysis demonstrated that adjunctive coiling contributed to mass effect relief in cavernous aneurysms, while dense embolism impeded symptom relief in aneurysms<10 mm and saccular aneurysms.

**Conclusions:**

Our data confirmed the efficacy of PED in relieving mass effect. The findings of this study provide support for endovascular treatment to alleviate mass effect in unruptured intracranial aneurysms.

**Trial registration number:**

NCT03831672.

**WHAT IS ALREADY KNOWN ON THIS TOPIC**

- The Pipeline Embolization Device (PED) has gained extensive popularity for its use in the treatment of patients with intracranial aneurysms. However, the efficacy of the PED in relieving the mass effect remains uncertain.

**WHAT THIS STUDY ADDS**

- Our data confirmed the efficacy of PED in relieving mass effect.

**HOW THIS STUDY MIGHT AFFECT RESEARCH, PRACTICE OR POLICY**

- This study provide support for PED to resolve the mass effect in unruptured intracranial aneurysms.

## Introduction

Unruptured intracranial aneurysms (UIAs) have the potential to induce symptoms through mass effect, which can result in cranial nerve palsies or compression of the brainstem.^1^ Flow diversion therapy has provided a novel approach to the treatment of intracranial aneurysms; it has achieved broad global acceptance over the last decade.^2 3^ The Pipeline Embolization Device (PED, Covidien/Medtronic, Irvine, CA) is extensively used for the treatment of intracranial aneurysms and has exhibited both safety and effectiveness across numerous large cohort studies.^4–7^ With advances in equipment and technology, UIA treatment aims not only to minimise the likelihood of aneurysm rupture and associated bleeding but also to relieve symptoms and improve the quality of life for patients. Several studies have reported the relief of the mass effect following the PED of UIA.^8–10^ However, the efficacy of the PED in relieving the mass effect remains a concern due to the limited sample size. Large, multicentre studies describing the effectiveness of PED for UIA patients presenting with mass effect are lacking. Thus, using data extracted from the large, multicentre, real-world cohort study, we have endeavoured to assess the effectiveness in alleviating the mass effect of UIA within the Chinese population, while also identifying potential predictors of treatment outcomes.

## Methods

### Study design and participants

The Post-Market Multi-Center Retrospective Research on Embolization of Intracranial Aneurysms with Pipeline Embolization Device in China (PLUS) Registry is a retrospective observational study with 14 participating centres across China from November 2014 to October 2019.^7 11 12^ The PLUS registry had specific inclusion criteria for participants, which included: (a) a diagnosis of an intracranial aneurysm using digital subtraction angiography (DSA), CT or MRI, regardless of the aneurysm’s shape or whether it was ruptured or unruptured; (b) treatment of the intracranial aneurysm with the PED. Any subjects who met any of the following exclusion criteria were not included: (a) treated with parent vessel occlusion; (b) participated in other embolisation devices; (c) lacked three-dimensional aneurysm images, or the images did not meet the simulation criteria.

This study aimed to assess the effectiveness of the PED in alleviating the mass effect due to intracranial aneurysms. Mass effect in this study refers to clinical or radiological signs that suggest a focal or global space-occupying effect resulting from a UIA, including but not limited to progressive headaches, nausea, vomiting, focal neurologic or radiologic findings (midline shift and/or herniation, oedema, and cranial nerve compression) without subarachnoid haemorrhage.^13^ Thus, after excluding 953 patients without mass effect, 218 patients were finally included in this study.

### Procedural details

Operators at each study centre used the PED at their discretion. Patients were treated with either the Classic PED or Flex PED, delivered and deployed through a Marksman microcatheter (Medtronic, Irvine, California). Coiling methodology was not specified in the protocol due to the retrospective nature of the study; the use of adjunctive coils was left to the operator’s preference and experience. Generally, the decision to use the PED in combination with coiling was made in situations where there was a potential risk of shortening and displacement of the device after release or when angiography revealed rapid blood flow at the aneurysmal neck. This combination approach was considered to mitigate the risk of recurrence and postoperative bleeding associated with using the PED alone.

Patients received antiplatelet therapy for a duration of 3 to over 6 months, typically combining aspirin (100 mg daily) and clopidogrel (75 mg daily). In cases of clopidogrel non-response, aspirin (100 mg daily) and ticagrelor (90 mg two times per day) were given. The preoperative dose of aspirin/clopidogrel was adjusted based on platelet function testing, performed consistently across all sites.

### Data collection and assessment

All patients were included in the index hospitalisation, which was defined as the first presentation and aneurysm repair at one of the registry centres for those with ruptured aneurysms and those with unruptured aneurysms without aneurysm repair. Clinical and radiological data were systematically recorded and documented at each study centre at index hospitalisation, discharge and follow-up assessments. The mass effect was evaluated by experienced neurologists, including nausea and vomiting with no other identifiable cause, and cranial neurological deficits (diplopia, vision impairment, visual field defect or dysphagia due to brain stem compression). The location of aneurysms can be categorised into two types: anterior circulation and posterior circulation. The former can be further classified into seven segments according to Shapiro *et al*.^14^ The latter includes aneurysms of the basilar artery, vertebral artery and other vessels of the posterior circulation. We defined unsuccessful device deployment as the failure of the PED to open or deployment of the PED inside the aneurysm. Successful device deployment after adjustments was defined as the successful release of the PED after technical adjustments. The PED was considered successfully deployed to the target site when it was released at the intended location without requiring any technical adjustments. Intraoperative angiography was used to evaluate the aneurysm occlusion rate. We assessed the treatment efficacy of aneurysms that received adjunctive coil embolisation using the Raymond-Roy Occlusion Classification (RROC) and coil packing density.^15^ Postoperative complications include mass effect deterioration, postoperative haemorrhagic stroke (mainly involving delayed aneurysm rupture or distal intraparenchymal haemorrhage) and postoperative ischaemic stroke or transient ischaemic attack (TIA).^16^ The improvement or deterioration of the mass effect was defined by patients’ symptoms. The neurological status of the patients was evaluated using the modified Rankin Scale Score.

Perioperative follow-up was conducted within 30 days postoperatively. Clinical and angiographic follow-up evaluations were conducted at specific time intervals, including 3, 6, 12, 24 and 36 months. Clinical follow-up was performed for all patients, regardless of imaging availability, and was conducted by telephone or email. The primary outcome was the mass effect relief at follow-up, assessed by neurological physical examination at outpatient follow-up or questionnaire. Aneurysm occlusion was assessed using DSA. During the follow-up, the patency of the parent artery was also evaluated via DSA.^17^ The first angiography image follow-up was performed between 3 and 6 months after PED implantation. Patients who demonstrated complete aneurysm occlusion on follow-up DSA did not require further routine angiographic follow-up. Otherwise, for those who did not achieve complete occlusion, additional angiographic evaluations were conducted for a period of up to 24 months or even longer.

A central review committee comprising three members, including a neurointerventionist, radiologist and neurosurgeon, was responsible for reviewing all the imaging and endpoint events. In situations where there were disagreements regarding the evaluation results, the committee engaged in a comprehensive discussion and reached a unanimous decision through consensus.

### Statistical analysis

All statistical analyses were performed using SPSS Statistics V.26.0 and R software V.4.1.3. P values were two sided, and p values<0.05 were considered significant.

Data are presented as the mean±SD for continuous variables and frequencies (percentage) for categorical variables. The original baseline differences between the whole cohort and patients with mass effect were evaluated using a t-test for continuous variables and a χ^2^ test for categorical variables. Univariate analysis was used to test covariates predictive of the mass effect relief at the last follow-up. Factors predictive on univariate analysis were entered into a multivariate logistic regression analysis.^18^ The ORs and 95% CIs of variables were calculated. To further determine the effect of adjunctive coiling and dense embolisms in different subgroups of UIA patients, we conducted subgroup analysis by aneurysm location, size and form. The cumulative mass effect relief rate was presented in Kaplan-Meier curves.

### Data availability

The data analysed in this study is governed by specific licenses and restrictions. To acquire access to the data, interested individuals should submit their proposals to the corresponding author for careful evaluation and consideration.

## Results

### Patients baseline

The PLUS registry included a total of 1171 patients with 1322 aneurysms who underwent treatment with the PED across 14 medical centres in China. In the present study, we enrolled 218 patients with mass effect treated with PED. Demographic and baseline characteristics of the whole cohort and patients presenting with mass effect were shown in table 1. Among patients presenting with mass effect, the average age was 54.3±11.8 years, and 74.0% (162/218) of patients were women. Comorbidities included hypertension (38.4%, 84/218), diabetes (4.1%, 9/218), hyperlipidaemia (2.8%, 6/218), cerebral infarction (4.6%, 10/218), cardiac disease (2.3%, 5/218), alcohol abuse (2.3%, 5/218) and smoking (14.6%, 32/218). No significant differences were observed in baseline characteristics between the whole cohort and patients with mass effect, except for onset symptoms. Aneurysms were unruptured but symptomatic. Cranial neurological deficit (such as diplopia, vision impairment, visual field defect, or dysphagia due to brain stem compression) was the most frequent (81.7%, 178/218) presentation, followed by nausea and vomiting (18.3%, 40/218).

**Table 1:** Baseline characteristics of patients presenting with mass effect

Characteristic	Total	Mass effect	P value
	N=1171	N=218	
Age, years	53.9±11.4	54.3±11.8	0.846
Sex			0.148
Male	358 (30.6%)	56 (25.6%)	
Female	813 (69.4%)	162 (74.0%)	
Smoking			0.339
Never	863 (73.7%)	167 (76.3%)	
Previous	91 (7.8%)	19 (8.7%)	
Current	217 (18.5%)	32 (14.6%)	
Alcohol abuse			0.454
Never	1027 (87.7%)	197 (90.0%)	
Previous	118 (10.1%)	16 (7.3%)	
Current	26 (2.2%)	5 (2.3%)	
Family history of aneurysm	18 (1.5%)	4 (1.8%)	0.746
Comorbidities			
Hypertension	397 (33.9%)	84 (38.4%)	0.187
Diabetes	63 (5.4%)	9 (4.1%)	0.444
Hyperlipidaemia	42 (3.6%)	6 (2.8%)	0.536
Cerebral infarction	54 (4.6%)	10 (4.6%)	0.987
Cardiac disease	58 (5.0%)	5 (2.3%)	0.083
Onset symptoms			
Incidental	425 (36.3%)	0 (0%)	<0.001
Symptomatic	704 (60.1%)	218 (100%)	
Nausea and vomiting	–	40 (18.3%)	
Cranial neurological deficit	–	178 (81.7%)	
Current SAH	42 (3.2%)	0 (0%)	
Patients with multiple aneurysms	260 (22.2%)	50 (22.8%)	
Total number of aneurysms treated with the Pipeline Embolization Device	1322	218	
Aneurysm size (maximum aneurysm length, mm)	12.79±8.75	19.60±10.36	0.658
<10 mm	630 (47.7%)	43 (19.7%)	0.001
10–25 mm	555 (42.0%)	115 (52.85%)	
>25 mm	137 (10.4%)	60 (27.5%)	
Aneurysm neck width, mm	6.21±3.92	8.96±7.46	0.335
Parent artery diameter, mm	3.88±0.82	3.94±0.76	0.059
Aneurysm form			0.071
Saccular	1099 (83.1%)	184 (84.4%)	
Fusiform	192 (14.6%)	34 (15.6%)	
Blister	31 (2.3%)	0 (0%)	
Location			0.355
Anterior circulation	1153 (87.2%)	195 (89.0%)	
Cavernous	269 (20.3%)	81 (37.5%)	
Paraophthlamic	707 (53.5%)	101 (46.3%)	
Posterior communicating and choroidal	111 (8.4%)	12 (5.5%)	
Terminus	18 (1.4%)	1 (0.6%)	
Anterior circulation distal	48 (3.6%)	0 (0%)	
Posterior circulation	169 (12.8%)	23 (11.0%)	
Basilar	29 (2.2%)	3 (1.4%)	
Vertebral artery and other vessels in the posterior circulation*	140 (10.6%)	20 (9.1%)	

Data are shown as n (%) or the mean±SD.

*Other vessels in the posterior circulation include the posterior cerebral artery and posterior inferior cerebellar artery.

SAH, subarachonoid hemorrhage.

### Aneurysm characteristics

Aneurysm characteristics are also presented in table 1. Out of the 268 aneurysms identified in the 218 patients, only 218 aneurysms were treated with PED. The mean aneurysm size was 13.04±9.76 mm, and the average neck size was 8.96±7.46 mm, respectively. The average parent artery diameter was 3.94±0.76 mm. Among the 218 aneurysms included in the study, 84.4% (184/218) were classified as saccular in morphology, while 15.6% (34/218) were categorised as fusiform. Most (89%, 195/218) of the aneurysms were located in the proximal anterior circulation. In comparison, 11.0% (23/218) of aneurysms were situated in the posterior circulation, with 1.4% (3/218) in the basilar artery and 9.1% (20/218) observed in the vertebral artery and other vessels within the posterior circulation. Compared with the whole cohort, aneurysms in patients with mass effect were more distributed in the 10 to 25 mm and >25 mm groups, while no significant difference was observed in other variables.

### Procedure characteristics

Treatment details are presented in table 2. The Classic PED and Flex PED were used in similar proportions (49.1%, 107/218 vs 50.9%, 111/218, respectively). Approximately 4.6% (10/218) of aneurysms were treated with multiple PEDs. Of 218 aneurysms, 41.7% (91/218) of aneurysms were treated with PED alone, while 58.3% (127/218) of aneurysms were treated with PED and coils. Among the cases that received adjunctive coiling, 22 (17.3%, 22/127) were classified as RROC I, 22 (17.3%, 22/127) as RROC II and 83 (65.4%, 83/127) as RROC III. Of these, 46 (36.2%, 46/127) aneurysms had a coil packing density greater than 90%, while 81 (63.8%, 81/127) had a packing density less than 90%. PEDs were deployed successfully in 203 (93.1%, 203/218) cases; 11 (5.1%, 11/218) were deployed successfully after adjustment, while 4 (1.8%, 4/218) failed to deploy.

**Table 2:** Treatment details and follow-up outcomes of patients presenting with mass effect

Characteristics	Frequency
	N=218 aneurysms
PED type	
Classic PED	107 (49.1%)
Flex PED	111 (50.9%)
Number of PEDs used	
Single	208 (95.4%)
Multiple	10 (4.6%)
Treatment modality	
PED only	91 (41.7%)
PED+coils	127 (58.3%)
Device deployment	
Unsuccessful	4 (1.8%)
Successful after adjustment	11 (5.1%)
Successful	203 (93.1%)
Satisfactory occlusion immediately after PED treatment	
Incomplete occlusion	188 (86.2%)
Complete occlusion	30 (13.8%)
Clinical outcomes	
Post-PED ischaemic stroke or transient ischaemic attack	14 (6.4%)
Post-PED haemorrhagic stroke	12 (5.5%)
Post-PED mass effect relief	73 (33.5%)
Post-PED mass effect deteriorate	21 (9.6%)
Parent artery occlusion	9 (4.1%)
Complete aneurysm occlusion at last follow-up	128 (58.7%)
Mass effect relief at follow-up	156 (71.6%)
mRS Score	
Pre-PED mRS Score	
0–2	216 (99.1%)
3–6	2 (0.9%)
Post-PED mRS Score (<30 days)	
0–2	204 (93.6%)
3–6	14 (6.4%)
mRS Score at follow-up	
0–2	207 (95.0%)
3–6	11 (5.0%)
Mortality	6 (2.8%)

Data are shown as n (%) or the mean±SD.

mRS, modified Rankin Scale; PED, Pipeline Embolization Device.

### Clinical and angiographic outcomes

**Figure 1:**
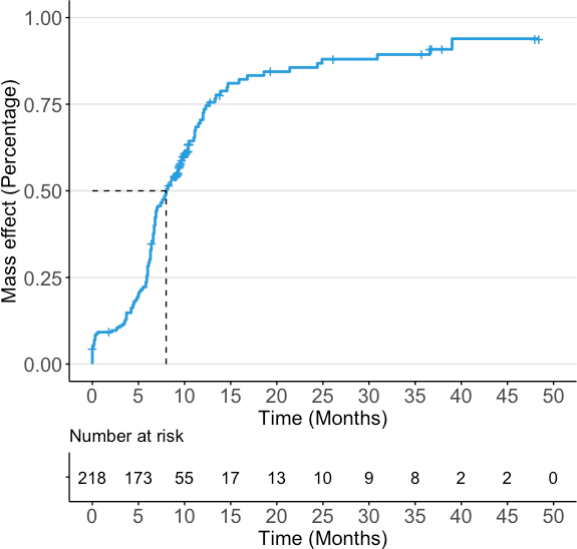
Kaplan-Meier curves showing cumulative rates of mass effect relief in the study cohort.

Ischaemic postoperative complications occurred in 14 patients (6.4%, 14/218) after PED deployment, 8 patients (3.7%, 8/218) were ischaemic stroke and 6 patients (2.8%, 6/218) were TIA. The post-PED haemorrhagic stroke rate was 5.5% (12/218). The mass effect was improved and deteriorated in 33.5% (70/218) and 9.6% (21/218) of patients immediately after surgery, respectively. Of 21 patients occurred mass effect deterioration, 7 patients (33.3%, 7/21) were treated with PED alone and 14 patients (66.7%, 14/21) received combined treatment of PED and coiling. Over a median follow-up period of 8.4 months (range from 1 to 48 months), the mortality rate observed was 2.8% (6/218). Nine (4.1%, 9/217) patients had complete occlusion of the parent artery during the follow-up period. Poor functional outcomes were observed in 6.4% (14/218) of patients during the early postoperative period and 5.0% (11/218) of patients during the follow-up periods, respectively. Approximately 71.6% (156/218) of patients were observed mass effect relief at the last follow-up. There was no significant differences in the mass effect relief rate between patients treated with PED alone (70.8%, 63/89) and PED plus coiling (73.2%, 93/127). With a sharp drop-off in mass effect proportion around the 6 months after treatment, the rate of mass effect relief approached 50% at approximately 8 months postoperatively (figure 1).

### Predictors of mass effect relief

**Figure 2:**
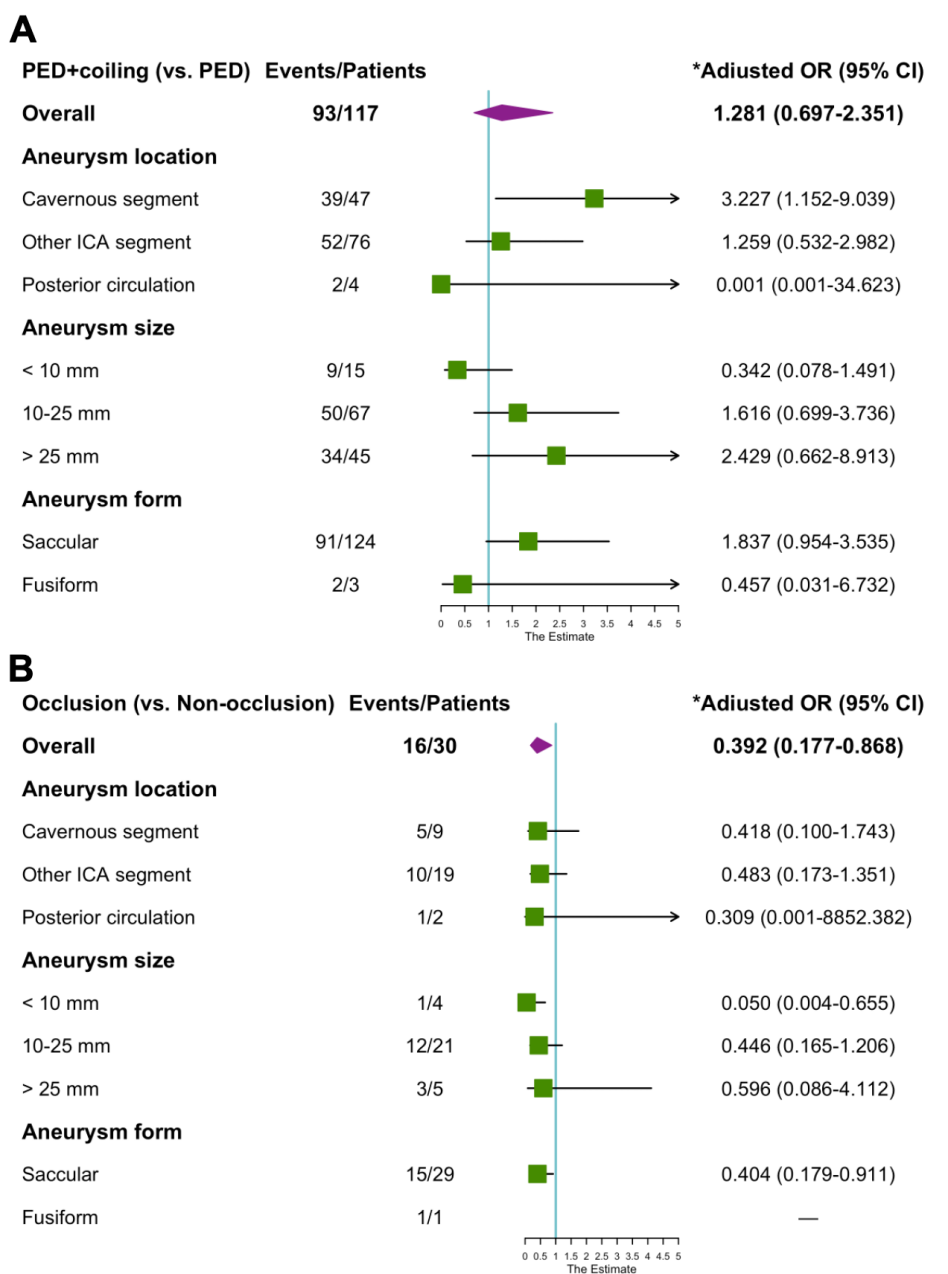
Forest plots portraying the ORs and 95% CIs for the associations between aneurysm factors and mass effect relief in patients with adjunctive coiling (A) and aneurysm occlusion immediately after treatment (B). Strata were aneurysm location (cavernous, ICA distal and posterior circulation), aneurysm size (<10, 10–25 and >25 mm) and aneurysm form (saccular or fusiform). *All models were adjusted for age and sex. PED, Pipeline Embolization Device. ICA, internal carotid artery.

On multivariate analysis, aneurysm occlusion immediately after PED treatment (OR 0.392, 95% CI, 0.170 to 0.907, p=0.029) was associated with mass effect relief at the last follow-up, while adjunctive coiling (OR 1.980, 95% CI, 0.990 to 3.958, p=0.053) did not reach statistical significance (table 3). The results of subgroup analysis by aneurysm location, size and form were presented in figure 2. Adjunctive coiling may help to alleviate the mass effect from cavernous aneurysms (OR 3.227, 95% CI, 1.152 to 9.039) whether the aneurysm is densely embolised intraoperative or not (OR 0.418, 95% CI, 0.100 to 1.743, while it was not significantly associated with any increased occurrence of mass effect relief in different aneurysm size and form subgroups. Furthermore, the aneurysm occlusion immediately after treatment was related to a lower mass effect relief rate in aneurysms less than 10 mm (OR 0.050, 95% CI, 0.004 to 0.655) and saccular form (OR 0.404, 95% CI, 0.179 to 0.911).

**Table 3:** Logistic regression analysis for promoting factors of mass effect relief at follow-up

Characteristics	Non-relief group	Relief group	Univariable	Multivariable	
	N=62	N=156	P value	OR (95% CI)	P value
Age, years	54.1±11.1	54.3±12.1	0.910	1.008 (0.981 to 1.036)	0.544
Female	48 (77.4%)	114 (73.1%)	0.508	0.665 (0.294 to 1.506)	0.722
Smoking	11 (17.7%)	40 (25.6%)	0.214		
Alcohol abuse	7 (11.3%)	14 (9.0%)	0.601		
Hypertension	23 (37.1%)	61 (39.1%)	0.784		
Diabetes	4 (6.5%)	5 (3.2%)	0.277		
Hyperlipidaemia	1 (1.61%)	5 (3.2%)	0.517		
Cranial neurological deficit	53 (85.5%)	125 (80.1%)	0.357		
Aneurysm size			0.995		
<10 mm	12 (19.4%)	31 (19.9%)	Ref	Ref	Ref
10–25 mm	33 (53.2%)	82 (52.6%)	0.930	0.908 (0.393 to 2.096)	0.821
>25 mm	17 (27.4%)	43 (27.5%)	0.983	0.891 (0.349 to 2.274)	0.809
Posterior circulation	3 (4.8%)	20 (12.8%)	0.084	1.622 (0.339 to 7.76)	0.545
Non-saccular form	5 (8.1%)	29 (18.6%)	0.053	2.636 (0.702 to 9.896)	0.151
Flex PED	26 (41.9%)	85 (54.5%)	0.094	1.653 (0.884 to 3.092)	0.116
Multiple PEDs used	2 (3.2%)	8 (5.1%)	0.545		
PED+coiling	34 (54.8%)	93 (59.6%)	0.519	1.980 (0.990 to 3.958)	0.053
Device unsuccessful deployment	2 (3.2%)	2 (1.3%)	0.335		
Post-PED mass effect deteriorate	7 (11.3%)	14 (9.0%)	0.601		
Intraoperative aneurysm occlusion	14 (22.6%)	16 (10.3%)	0.017	0.392 (0.170 to 0.907)	0.029

PED, Pipeline Embolization Device.

## Discussion

The findings from the PLUS registry provide evidence supporting the safety and efficacy of the PED for the treatment of UIA in the Chinese population^7 11^; the rates of complete occlusion, complications and mortality are comparable to those reported in Western populations.^16 19^ Of note, our results confirm that the PED can also effectively relieve the symptoms in patients presenting with mass effect. In further analysis, we found that aneurysm occlusion immediately after treatment is opposed to mass effect relief at the follow-up. Moreover, the mass effect relief may benefit from adjunctive coiling, whether the aneurysm is densely embolised intraoperative or not for cavernous aneurysms. Conversely, for aneurysms<10 mm and saccular aneurysms, immediate aneurysm occlusion after treatment may be detrimental to mass effect relief at follow-up.

The improved quality of intracranial imaging technologies has led to the increased detection of UIAs. Occasionally, UIAs can be incidentally detected during imaging performed for unrelated reasons, as they grow and exert mass effect on adjacent central nervous system structures.^20^ Such mass effect includes third cranial nerve palsy associated with posterior communicating artery or basilar artery aneurysms; cavernous sinus syndrome caused by cavernous sinus aneurysms; hemiparesis, field defects or seizure related to middle cerebral artery aneurysms; and compression of the brainstem owing to basilar distribution aneurysms. Additionally, other cranial nerves such as the trochlear and abducens nerves, as well as the first division of the trigeminal nerve, can be involved. The mass effect rate (18.6%, 218/1171, 95% CI, 0.165 to 0.210) in the PLUS registry cohort is higher than that reported by several studies in the western and Japanese populations.^21 22^ It may be because the two previous cohorts included conservatively treated patients, whereas the patients in this cohort are interventional treated. Thus, the aneurysmal symptoms are more frequently observed in the PLUS registry. Our data demonstrated that large and giant UIAs are more vulnerable to mass effect. Consistent with the view that that symptoms related to aneurysmal mass effect are infrequently observed in small aneurysms,^23^ 6.8% (43/630) of patients with UIAs smaller than 10 mm present with neurologic symptoms in our cohort. These symptoms are typically arise from the compression of the second and third cranial nerves, consistent with the previous study.^24^ Given the frequency with which this event occurs, the clinician must consider the possibility of unilateral visual acuity loss due to mass effect from a small UIA in the differential diagnosis of any patient with field defect.

The occlusion rate observed in the entire PLUS cohort (81.4%, 787/967) is similar to the rates reported by previous studies.^11^ For instance, the Pipeline for Uncoilable or Failed Aneurysms study documented a 12-month follow-up complete occlusion rate of 86.8%.^5^ Similarly, in a single-centre study comprising 445 cases of anterior circulation aneurysms treated with PED, a follow-up period of 14 months revealed a complete occlusion rate of 82%.^25^ However, the study cohort exhibited a complete occlusion rate of approximately 60% during the follow-up period. This discrepancy in the rate of complete occlusion may be attributed to the larger size of aneurysms in the study cohort and the relatively shorter mean follow-up time of only 8 months. Furthermore, the utilisation of a single device in the majority of aneurysms may have decreased the likelihood of achieving complete occlusion.^26^ However, it is important to note that the use of multiple devices may also carry an increased risk of complications such as thromboembolism, vessel perforation and aneurysm rupture.^27 28^ Thus, the decision to use multiple devices should be made cautiously on a case-by-case basis, taking into account the aneurysm characteristics, patient factors and operator experience.

The effectiveness of the PED in reducing of aneurysmal mass-effect symptoms remains controversial. Patel *et al* reported a recovery of visual function in a patient with bilateral visual loss caused by a giant ophthalmic aneurysm after flow diversion embolisation. The improvement in vision was due to both reduction in mass effect and aneurysm pulsation.^29^ In a small series involving 27 patients with 30 aneurysms larger than 10 mm, who were treated exclusively with flow diversion, the study reported that 94% (16/17) of patients experienced either improvement or complete relief from mass-effect symptoms after the procedure.^10^ However, these results may not be generalisable or representative of the overall population due to the limited sample size. Thus, this multicentre study on a high-volume cohort of UIAs with mass effect can provide more information on the effectiveness of PED in relieving mass effect. In the present study, the mass effect was improved in 71.6% (156/218) of patients at the follow-up. The thrombogenicity of the dense coil mass has been reported to inhibit the mass effect relief. In this study, it also appears that the coil mass affects the immediate symptom relief after treatment and the long-term mass effect relief, especially for aneurysms<10 mm and saccular aneurysms. In the present study, the proportion of mass effect relief increased sharply at 6 months postoperatively. It somewhat indicates significant changes in haemodynamics 6 months after PED treatment. Thus, the duration of dual-antiplatelet therapy over 6 months may be essential to avoid the formation of microemboli due to haemodynamic alterations.

Considering the series is very diverse regarding aneurysm location, size and form. Mass effect from cavernous aneurysms causing cranial neuropathies may differ from intradural posterior communicating artery aneurysms causing mass effect with third nerve compression and fusiform posterior circulation aneurysms causing mass effect on the cerebellum. We dissect these data into more specific, less broad categories with regard to location, size and form of aneurysms. Interestingly, subgroup analysis demonstrated that adjunctive coiling helps to mass effect relief in cavernous aneurysms. Most cavernous aneurysms (86.9%, 73/84) were large or giant aneurysms; PED plus adjunctive coiling can accelerate intraluminal thrombus formation and organisation process. The faster the aneurysm collapse the shorter the cranial nerve is compressed. Thus, the mass effect is more likely to relieve. Although immediate aneurysm occlusion was not significantly related to mass effect relief in some subgroups, dense embolisation seems not to be a good idea. The dense coil mass could transform a pliable pulsation mass into a firm structure and transfer the arterial pulsation from the aneurysm wall to the adjacent tissue, potentially intensifying or inducing mass effect.^10^ Similar to our results, immediate aneurysm occlusion was associated with a lower mass effect relief rate, especially in small and saccular aneurysms.

Another important issue is the deterioration of the mass effect immediately after the PED treatment. In the present study, mass effect deterioration occurred in 21 (9.6%, 21/218) patients in the early postoperative period, which was rarely observed during the follow-up period. The adjunctive coiling also leads to a higher deterioration rate in patients treated with PED plus coiling. Most patients (66.7%, 14/21) who occur postoperative mass effect deterioration had mass effect relief during later follow-up. Although the aneurysm’s volume may become significantly larger in the early stage of thrombosis, which often leads to mass effect deterioration. The mass effect will decrease with the thrombus organisation and the collapse of the aneurysm after several weeks.

### Limitations

This study includes a robust high-volume cohort of UIA patients with mass effect and can provide some information about the effectiveness of PED in relieving mass effect. However, some study limitations must be addressed to interpret our data accurately. First, the retrospective design of the study and the variations in management and PED treatment across different centres may introduce potential biases. Also, the relatively short median follow-up time of 8.4 months may not be sufficient for complete aneurysm occlusion, leading to underestimating the actual mass effect relief rate.

## Conclusions

The observed high rate of symptom improvement in PED-treated UIA patients proves the practicality and validity of the PED in treating UIA patients presenting with mass effect. Patients with immediate aneurysm occlusion showed a lower rate of mass effect relief than those without. Also, adjunctive coiling helps to mass effect relief in cavernous aneurysms. However, dense embolisation was not suggested, especially for small and saccular aneurysms. The results of this study provide support for endovascular treatment to resolve the mass effect in UIA.

## Data Availability

Data are available upon reasonable request.
